# Advocacy as an Academic and Nurse Practitioner

**DOI:** 10.1093/geroni/igaa057.2383

**Published:** 2020-12-16

**Authors:** Lori Martin-Plank

**Affiliations:** University of Arizona, Pipersville, Pennsylvania, United States

## Abstract

The first speaker is Dr. Lori Martin-Plank, an established academic at the University of Arizona, College of Nursing. Dr. Martin-Plank will provide her experiences in advocating for older adults in Pennsylvania and nationally through professional organizations, meeting with coalition partners to promote access to care for vulnerable older adults in rural areas by promoting full practice authority for nurse practitioners, and advocating for full home health authority for nurse practitioners. Dr. Martin-Plank will share how she is active in advocacy and policy at the local, state and federal levels, and how to build a presence and relationship with legislators on The Hill and State Capitol. Dr. Martin-Plank is a family, gerontological, and mental health nurse practitioner, practicing in Pennsylvania, New Jersey, and Arizona.

